# Patient‐Centered Teledermatology: Bridging Pharmacies and Dermatologists

**DOI:** 10.1155/ijta/9409013

**Published:** 2026-07-29

**Authors:** Nuria Perez-Cullell, Valérie Buresi, Nicolas Homehr, Jean-Ludovic Brutin, Julie Fraysse

**Affiliations:** ^1^ Direction Médicale et Relation Patient/Consommateur, Pierre Fabre Laboratories, Toulouse, France; ^2^ Private Practice, Lherm, France; ^3^ Department of Dermatology, Centre Hospitalier Marie-Josée Treffot, Hyères, France; ^4^ Pharmacie de l′Europe, Maurs, France

## Abstract

**Background:**

The chronic shortage of dermatologists in France results in average waiting times of 3 months, compromising equitable access to care and early diagnosis of severe conditions such as skin cancers. This pilot project evaluates the feasibility, effectiveness, and acceptability of a tele‐expertise platform connecting community pharmacists and dermatologists.

**Methods:**

From March 2024 to March 2025, 40 pharmacies (18 urban and 22 rural) submitted 731 dermatological cases via a secure platform, following pharmacist training in clinical photography and best practices. Twelve volunteer dermatologists responded within a target of 24–48 h. Data focused on utilization, response times, pathologies, satisfaction (pharmacist surveys and pharmacist‐reported patient feedback), and qualitative feedback from an expert board.

**Results:**

Seven hundred fourteen tele‐expertises were completed, with a mean response time of 5.4 days, reflecting early implementation constraints and progressively improving over time (reduced to 1–2 days by study end; 83% < 48 h in February 2025). The main conditions include suspected melanoma (7), carcinomas (27), actinic keratosis, eczema, atopic dermatitis, rosacea, vitiligo, and shingles. Pharmacist satisfaction reached 72% (NPS 24%; 94% recommending a scale‐up with adaptations). Pharmacists reported that 88% of patients were satisfied. Limitations comprised the lack of e‐prescriptions and the nonintegration of primary care physicians.

**Conclusion:**

This pharmacy‐centered model improves rapid access to dermatological expertise, reduces diagnostic delays, and satisfies users, but requires integration into coordinated care pathways (with primary care physicians), pharmacist remuneration, and legal/financial reforms for sustainability. A second pilot is testing these recommendations.

## 1. Introduction

Teledermatology (TD) is most commonly practiced in the form of teleconsultations between patients and dermatologists. However, it also encompasses tele‐expertise (TE) exchanges between healthcare professionals. The most widely recognized application of TE involves primary care physicians consulting dermatologists to optimize diagnosis and management [1]. Beyond this well‐established model, TE interactions can also occur between pharmacists and dermatologists, particularly in contexts where pharmacists are frontline advisors for patients with dermatological concerns [2].

The chronic shortage of dermatologists in France represents a major public health challenge, with average waiting times of 3 months for a specialist appointment [3]. This situation compromises equitable access to care and delays the diagnosis of critical conditions such as skin cancers [4]. In response to this shortage, innovative solutions leveraging telemedicine and interprofessional collaboration are essential to rethink care pathways. Community pharmacies, with their dense territorial network and presence in almost every French municipality, [5] represent a strategic choice to facilitate access to dermatological expertise. This pilot project evaluates the feasibility, effectiveness, and acceptability of a TE platform enabling pharmacists to transmit dermatological cases to specialists, with the aim of reducing diagnostic delays and laying the foundations for a sustainable model to improve access to specialized care.

## 2. Methods

This pilot study was conducted over a 1‐year period, from March 2024 to March 2025, with the primary aim of evaluating the feasibility, effectiveness, and user satisfaction of a TE service for dermatological cases submitted by community pharmacists. The study involved 40 pharmacies distributed across various geographic and demographic settings: 18 were located in urban areas, whereas 22 were in rural regions. The participating pharmacies varied in size, including 8 small, 20 medium, and 12 large or very large establishments, reflecting a representative mix of pharmacy environments.

The service was supported by 12 volunteer dermatologists (both private and hospital practitioners, based in France) who provided expert assessments and guidance. Prior to the study, pharmacists received specific training focused on good practices in TE, the clinical photography of skin conditions, and the use of the TE digital platform, ensuring standardized and high‐quality case submissions.

Pharmacists submitted cases through a secure online platform by uploading clinical photographs and completing a structured clinical information form. Cases were expected to be reviewed and responses provided by the dermatologists within a target window of 24–48 h. The scope of the study excluded certain categories, such as medical emergencies, mucosal pathologies, nonevolving scars, and recent warts, to focus on dermatological conditions most amenable to remote evaluation and timely specialist input.

The data collected throughout the pilot were analyzed along three main dimensions:

**1. TE Utilization and Clinical Outcomes**

This included detailed statistics on the number of TE requests submitted, the types of dermatological conditions assessed, the response times from dermatologists, and notable diagnostic outcomes such as suspected cases of melanoma and carcinoma. The progression of response times over the study period was tracked to assess improvements in efficiency.

**2. User Experience and Satisfaction Surveys**

**Figure 1:**
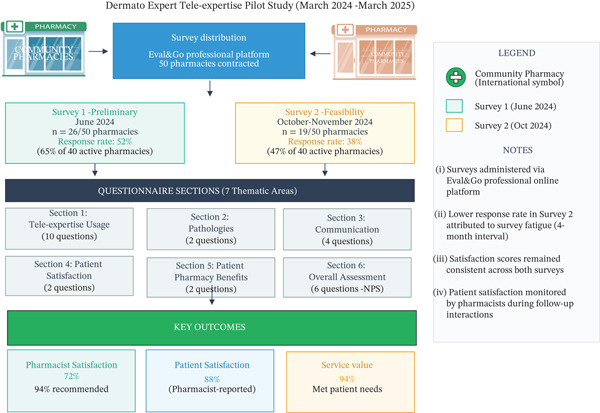
Pharmacist survey administration flowchart showing the distribution and collection process across participating pharmacies.

Two separate pharmacist surveys were conducted using Eval&Go, a professional online survey platform, to assess the feasibility and acceptability of the TE system (Figure 1). Structured questionnaires were distributed by email. The first survey, a preliminary assessment in June 2024, gathered initial feedback with 26 respondents out of 50 contracted pharmacies (52% response rate, or 65% of the 40 actively participating pharmacies). A more comprehensive feasibility survey was performed in October–November 2024, with 19 respondents out of 50 (38% response rate, or 47% of active pharmacies). The lower response rate in October can be attributed to survey fatigue, as we had already conducted an initial survey only 4 months earlier. Importantly, the results and trends (particularly satisfaction rates) were consistent between both surveys, suggesting representative data. Some pharmacies likely did not respond because they had few or no eligible cases during the study period, reducing the perceived relevance of completing the survey. These surveys collected data on usage frequency, time spent per TE session, satisfaction levels, perceived benefits, and limitations. Patient satisfaction was monitored by pharmacists during follow‐up interactions and reported as part of the pharmacist survey responses.

**3. A Board of Medical Experts**

The board comprised one primary care physician and four dermatologists who were questioned about their qualitative feedback on dermatological TE and operational barriers.

Key performance indicators were defined a priori and included the volume of TE requests, mean response times, spectrum and frequency of diagnosed pathologies, pharmacist satisfaction scores, pharmacist‐reported patient feedback, and qualitative feedback on operational barriers. These metrics were used to evaluate the clinical and operational feasibility of the pilot, as well as to identify necessary adaptations for scaling the program to a broader population.

## 3. Results

A total of 731 TE requests were submitted by pharmacists between March 2024 and March 2025, with 714 completed during the pilot period. The mean response time was 5.4 days (130.3 h), progressively decreasing to 1–2 days by February–March 2025, when 57% of TE requests were resolved within 48 h (rising to 83% in February 2025). Regarding the pharmacist surveys: among 50 pharmacies contacted, 26 participated in the preliminary survey conducted in June 2024 (52% response rate, or 65% of active pharmacies), and 19 participated in the feasibility survey conducted in October–November 2024 (38% response rate, or 47% of active pharmacies). The difference in response rates between surveys is attributable to survey fatigue following the initial assessment 4 months earlier. Despite this difference, satisfaction scores and reported trends remained consistent across both surveys.

Pharmacists most frequently submitted TE requests for suspected melanoma, carcinoma, actinic keratosis, eczema, atopic dermatitis, rosacea, vitiligo, and shingles. Notable successes included seven suspected melanomas, 27 carcinomas (one surgically treated within 72 h), and rapid management of rosacea and vitiligo cases within 48 h.

The survey of pharmacists included 19 respondents (RR = 38*%*). On average, pharmacists performed two TEs per week, each lasting about 15 min when done in the pharmacy. Pharmacists reported an overall satisfaction rate of 72%, with 94% recommending scale‐up (47% with adaptations, such as pharmacist remuneration, platform optimization, and recruiting more dermatologists for better territorial coverage).

Regarding recommending the service to a colleague, pharmacists gave an average score of 7.6 out of 10, corresponding to a net promoter score (NPS) of 24%.

When asked if they would be willing to pay for the solution after the pilot phase, 31% answered yes.

Around 68% of them found the platform navigation to be satisfactory or very satisfactory, and 89% expressed a desire for a mobile app to improve ease of use. Following the TE, the main actions taken were providing medical advice and pharmacy counseling or referring patients to their primary care doctor or another specialist.

Patients also expressed high satisfaction (88% in June 2024: 53% of patients reported being very satisfied, and 35% were satisfied), though 50% pointed out the absence of direct prescriptions.

## 4. Discussion

A report by the General Inspectorate of Finance (IGF) and the General Inspectorate of Social Affairs (IGAS) noted that in 2016, 97% of the French population lived less than 10 min by car from a pharmacy, and 99.5% lived less than 15 min [5].

This geographic accessibility positions pharmacies as strategic access points in both the healthcare delivery system and the healthcare patient journey, enabling faster access to dermatological expertise and enhancing patient care experiences, particularly in underserved areas.

The TE pilot involving pharmacists tapped into this potential by enabling pharmacists to submit dermatological cases remotely to specialists for expert evaluation. The types of cases submitted ranged widely, from suspicious pigmented lesions such as possible melanomas or carcinomas to inflammatory and chronic skin conditions including eczema, atopic dermatitis, rosacea, vitiligo, and shingles [6–8].

Pharmacists were able to obtain expert dermatological advice in a timely manner, which led to the diagnosis of significant pathologies, including malignant skin tumors that required urgent management. This illustrates that pharmacists can play a role in the early identification of severe conditions, facilitating faster referrals and treatment. For patients, the convenience of accessing specialist input through their local pharmacy without the need for immediate travel to a hospital or dermatologist′s office was highly appreciated. Patient satisfaction was generally high, particularly regarding the overall speed and responsiveness of the service. Pharmacists also reported substantial satisfaction with the TE system, noting its potential to improve patient care and their own clinical practice.

Beyond issues of specialist availability alone, it is important to clarify why pharmacist‐to‐dermatologist TE offers distinct structural and clinical advantages. First, pharmacists serve as trained healthcare professionals who can perform initial triage, ensuring that appropriate cases are submitted for specialist review and reducing the burden on dermatologists from inappropriate referrals. Second, trained pharmacists take higher quality clinical photographs following standardized protocols, compared with patient‐submitted images that often suffer from poor lighting, focus, or framing. Third, pharmacists collect a comprehensive clinical history using structured forms, enabling dermatologists to make more informed assessments. Fourth, pharmacists can relay and explain the dermatologist′s recommendations, ensuring patients understand their diagnosis and treatment plan. Finally, this model optimizes dermatologists′ time by reserving in‐person consultations for complex, doubtful, or high‐risk cases. The service is primarily utilized for patients experiencing difficulty accessing either primary care physicians or dermatologists, or when healthcare professionals deem the scheduled medical appointment too delayed relative to clinical urgency. However, it represents more than a solution to availability issues—it is an optimized care pathway that decongests medical practices while providing structured, secure responses for cases that do not require physical consultation.

However, these positive outcomes must be interpreted with caution, recognizing the inherent complexities and limitations of TD when implemented outside of fully integrated healthcare networks [9, 10]. While diagnosing a suspicious lesion remotely is valuable, it is only the first step in a continuum of care that must include confirmatory diagnostics, multidisciplinary assessment, and coordinated treatment planning [11]. Skin cancers, for instance, require multidisciplinary tumor boards to review cases, establish consensus on diagnosis, and define the therapeutic approach. Without strong connections to hospital services and established care pathways, remote diagnoses made by private dermatologists may not translate into timely or appropriate management [12–14].

Furthermore, the pilot exposed significant structural barriers that currently hinder the scalability and sustainability of TD in pharmacy settings. One major issue is the lack of financial recognition and remuneration for pharmacists who dedicate their time and expertise to TE activities. Without adequate compensation, pharmacists may find it difficult to maintain or expand their involvement, limiting the long‐term viability of such programs. Legal constraints also prevent physicians from dispensing an e‐prescription after a TE, which reduces the clinical impact of the service and can prolong the patient journey by requiring additional consultations.

Regarding the regulatory context of pharmacy practice in France, pharmacists currently have limited prescribing authority. Although recent regulatory changes have expanded certain vaccination and minor ailment management roles, pharmacists cannot independently prescribe dermatological treatments requiring medical prescription. Facilitating pharmacist prescribing was not an objective of this study, which was primarily focused on early screening and improving access to dermatological care. Furthermore, our survey results indicate a relatively low percentage of pharmacies that believed TE would significantly increase their prescribing activity. The primary value identified by participating pharmacists was in responding to unmet patient needs for specialist access, rather than expanding their prescribing role.

Another notable limitation is the insufficient integration of primary care physicians into the TE workflow. Primary care doctors often remain uninformed of TE results unless manually notified by the teledermatologist, breaking the chain of communication that is essential for coordinated care. This lack of automatic information sharing between TE platforms and existing healthcare programs represents a missed opportunity to streamline patient management and ensure continuity of care. Without seamless digital interoperability, patients risk becoming lost between different providers, undermining the benefits of rapid specialist input.

Addressing these challenges requires a multifaceted approach [14–16]. The medical advisory board recommends reintegrating primary care physicians into the TE process from the outset, ensuring they receive timely notifications and can actively participate in patient follow‐up. Establishing pools of salaried dermatologists within multiprofessional health centers, particularly in underserved regions, would provide more stable and equitable access to specialist expertise, improving territorial coverage and reducing regional disparities in dermatological care. To implement these recommendations, a second pilot project is currently underway to test the integration of primary care physicians into the TE workflow and explore remuneration models for pharmacists. The results of this second pilot, to be published in a future study, are expected to provide further insights into enhancing the efficiency and sustainability of the pharmacy‐based TD model.

Moreover, significant policy reforms will be necessary to support TD′s expansion. This includes defining clear reimbursement models for pharmacists′ TE activities to encourage sustained engagement, integrating TD into existing health pathways, and securing institutional backing from professional dermatology organizations. Comprehensive economic evaluations should be conducted to assess cost‐effectiveness, guide funding decisions, and demonstrate the value of TD as a component of modern healthcare.

While this pilot shows that pharmacists can indeed contribute meaningfully, it also reinforces that such initiatives cannot operate in isolation. TD must not become a fragmented solution but should instead be integrated within a coordinated healthcare system.

## 5. Conclusion

In conclusion, this pilot project underscores the promising potential of pharmacies to enhance access to dermatological expertise, particularly in underserved regions, while reducing diagnostic delays and improving patient satisfaction. However, it also highlights the need for closer integration into coordinated care pathways involving multidisciplinary teams, hospital networks, and primary care providers. To address these challenges, a second pilot is currently underway to test the recommendations of the medical advisory board, particularly by strengthening collaboration with general practitioners and exploring sustainable funding models. The results of this second pilot, to be published in a future study, are expected to provide further evidence to establish TD as a sustainable pillar of dermatological care. Only through such integration, supported by legal, financial, and technological reforms, can TD fully realize its potential within a coordinated healthcare system. Importantly, our project focuses on the expertise of dermatologists rather than AI. This approach meets the requirements of a rigorous ethical framework, guaranteeing fair and secure access to dermatological care, as recommended by learned societies such as the French Society of Dermatology (SFD) [17].

## Funding

This study was supported by Les Laboratories Pierre Fabre (10.13039/100013226).

## Ethics Statement

I/we confirm that the study was conducted in accordance with applicable ethical standards. As this project was a feasibility pilot based on anonymized clinical photographs and pharmacist‐mediated tele‐expertise, formal IRB approval was not required under French regulations. All participating pharmacists obtained informed consent from patients prior to case submission. This study did not require formal Institutional Review Board approval under French law, as it involved anonymized tele‐expertise data collected in a pilot healthcare service evaluation. All patients provided informed consent prior to participation.

## Conflicts of Interest

Nuria Perez‐Cullell and Valérie Buresi are employed by Pierre Fabre. Other authors declare no conflicts of interest.

## Supporting information


**Supporting Information** Additional supporting information can be found online in the Supporting Information section. Complete survey questionnaire administered to pharmacists, including all questions on sun protection practices, product recommendations, and counseling patterns.

## Data Availability

The datasets generated and analyzed during the current study are not publicly available due to privacy restrictions but are available from the corresponding author on reasonable request.
