# Supplementary material for: Patient‐Centered Teledermatology: Bridging Pharmacies and Dermatologists
Source: Int J Telemed Appl. 2026 Jul 29;2026:9409013. doi: 10.1155/ijta/9409013 (PMC13417487; doi:10.1155/ijta/9409013)
Supplement: Supplementary file 1 — Supporting Information Additional supporting information can be found online in the Supporting Information section. Complete survey questionnaire administered to pharmacists, including all questions on sun protection practices, product recommendations, and counseling patterns. [file IJTA-2026-9409013-s001.docx]

# **Supplementary Material**

*Pharmacist Survey Questions*

*Patient-Centered Teledermatology: Bridging Pharmacies and Dermatologists*

This supplementary material provides the survey questions used to assess pharmacist satisfaction and experiences with the teledermatology pilot program. Two surveys were conducted: a preliminary survey in June 2024 (Survey 1) and a comprehensive feasibility survey in October 2024 (Survey 2). Both surveys were administered via Eval&Go, a professional online survey platform. Questions are presented in their original French with English translations.

## Section 1: Tele-expertise Usage and Platform

**Q1.1:** *A titre individuel combien de téléexpertises avez-vous réalisées en moyenne par semaine ?*
[How many tele-expertises have you personally performed on average per week?]
**Response type:** Numeric (open field) | **Included in:** Both

**Q1.2:** *Combien de temps dure en moyenne la réalisation d'une téléexpertise en officine (incluant, dossier patient, questionnaire et prise de photos) ?*
[How long does a tele-expertise session typically take in the pharmacy (including patient file, questionnaire, and photos)?]
**Response type:** Numeric (minutes) | **Included in:** Both

**Q1.3:** *Le délai de réponse des dermatologues vous semble-t-il adapté aux besoins de vos patients ?*
[Does the dermatologists' response time seem adequate for your patients' needs?]
**Response type:** Satisfaisant / Trop long / Pas du tout (Satisfactory / Too long / Not at all) | **Included in:** Both

**Q1.4:** *Comment trouvez-vous la navigation sur la plate-forme DermatoExpert ?*
[How do you find the navigation on the DermatoExpert platform?]
**Response type:** Scale 1-3 (1=Difficult, 3=Easy) | **Included in:** Both

**Q1.5:** *Souhaitez-vous partager des propositions d'amélioration de la plate-forme ?*
[Do you have any suggestions for platform improvements?]
**Response type:** Open text | **Included in:** Survey 2

**Q1.6:** *Souhaiteriez vous la mise à disposition d'une application mobile pour la réalisation d'une téléexpertise sur smartphone ou tablette ?*
[Would you like a mobile application for performing tele-expertise on smartphone or tablet?]
**Response type:** Oui / Non (Yes / No) | **Included in:** Survey 2

**Q1.7:** *Avez-vous rencontré des difficultés dans la lecture et la retranscription des comptes rendus des dermatologues ?*
[Have you encountered difficulties in reading and transcribing dermatologists' reports?]
**Response type:** Oui / Non (Yes / No) | **Included in:** Both

**Q1.8:** *Si oui pour quelles raisons ?*
[If yes, for what reasons?]
**Response type:** Open text (conditional) | **Included in:** Both

**Q1.9:** *Quelles suites de téléexpertises les plus courantes avez vous pu observer ?*
[What are the most common outcomes you have observed following tele-expertise?]
**Response type:** Ranking 1-4: Avis médical et conseil / Réorientation vers le médecin traitant / Proposition de téléconsultation / Proposition de consultation physique | **Included in:** Both

**Q1.10:** *Avez-vous observé d'autres pratiques à la suite d'une téléexpertise ?*
[Have you observed other practices following a tele-expertise?]
**Response type:** Open text | **Included in:** Both

## Section 2: Pathologies

**Q2.1:** *Classez les pathologies les plus fréquentes pour lesquelles vous avez fait une téléexpertise (1 la plus fréquente 5 la moins fréquente)*
[Rank the most frequent pathologies for which you performed tele-expertise (1=most frequent, 5=least frequent)]
**Response type:** Ranking: Mélanome/carcinome suspicion, Kératose actinique, Eczéma de contact, Dermatite atopique, Rosacée, Traumatique, Acné, Psoriasis, Dermatite séborrhéique, Vitiligo, etc. | **Included in:** Both

**Q2.2:** *Avez-vous traité de manière fréquente d'autres pathologies en téléexpertise ?*
[Have you frequently treated other pathologies via tele-expertise?]
**Response type:** Open text | **Included in:** Both

## Section 3: Communication and Training (Survey 1)

**Q3.1:** *Avez-vous installé la PLV ?*
[Have you installed the point-of-sale materials?]
**Response type:** Oui / Non (Yes / No) | **Included in:** Survey 1

**Q3.2:** *Avez-vous communiqué sur vos réseaux sociaux sur ce nouveau service ?*
[Have you communicated about this new service on your social media?]
**Response type:** Oui / Non (Yes / No) | **Included in:** Survey 1

**Q3.3:** *Souhaiteriez-vous mettre en place des thématiques de dépistages (la semaine du mélanome, la semaine de l'eczéma,...) ?*
[Would you like to implement screening themes (melanoma week, eczema week, etc.)?]
**Response type:** Oui / Non (Yes / No) | **Included in:** Survey 1

**Q3.4:** *Vos patients ont connu le service DermatoExpert de votre officine via :*
[How did your patients learn about the DermatoExpert service at your pharmacy?]
**Response type:** Votre conseil / la PLV / Réseaux sociaux / Bouche à oreille | **Included in:** Survey 1

## Section 4: Patient Satisfaction

**Q4.1:** *Quel est le niveau de satisfaction de vos patients ?*
[What is your patients' satisfaction level?]
**Response type:** Scale 1-4 (1=Very dissatisfied, 4=Very satisfied) | **Included in:** Both

**Q4.2:** *Quels sont les retours de vos patients sur ce nouveau service ?*
[What feedback have you received from your patients about this new service?]
**Response type:** Open text | **Included in:** Both

## Section 5: Pharmacy Benefits

**Q5.1:** *D'un point de vue officine, ce service vous a permis de:*
[From a pharmacy perspective, this service has allowed you to:]
**Response type:** Multiple choice: Répondre à un besoin patient / Fidéliser votre patientèle / Acquérir de nouveaux patients / Vendre des produits supplémentaires / Faire monter en compétence vos collaborateurs | **Included in:** Both

**Q5.2:** *Pouvez-vous nous partager un ou deux succès, cas qui vous ont marqués ?*
[Can you share one or two success stories that stood out to you?]
**Response type:** Open text | **Included in:** Both

## Section 6: Overall Assessment and Recommendations

**Q6.1:** *Quelles seraient vos 3 principales recommandations à de nouvelles pharmacies pour un service réussi ?*
[What would be your 3 main recommendations for new pharmacies for a successful service?]
**Response type:** Open text | **Included in:** Both

**Q6.2:** *Recommandez-vous le déploiement du service en France ?*
[Do you recommend scaling up the service in France?]
**Response type:** Oui tout à fait / Non / Oui mais avec des adaptations (Yes definitely / No / Yes but with adaptations) | **Included in:** Both

**Q6.3:** *Précisez pourquoi (principales difficultés, améliorations nécessaires)*
[Please specify why (main difficulties, necessary improvements)]
**Response type:** Open text (conditional) | **Included in:** Both

**Q6.4:** *Quel est votre niveau de satisfaction globale ?*
[What is your overall satisfaction level?]
**Response type:** Scale 1-4 (1=Very dissatisfied, 4=Very satisfied) | **Included in:** Both

**Q6.5:** *Recommanderiez vous ce service à un confrère ? (0 pas du tout, 10 tout à fait)*
[Would you recommend this service to a colleague? (0=Not at all, 10=Absolutely)]
**Response type:** NPS Scale 0-10 | **Included in:** Both

**Q6.6:** *Quelles sont les bonnes pratiques que vous avez mis en place dans votre pharmacie que vous souhaiteriez partager ?*
[What best practices have you implemented in your pharmacy that you would like to share?]
**Response type:** Open text | **Included in:** Survey 1

## Section 7: Commercial Considerations (Survey 2 only)

**Q7.1:** *A l'issue du pilote l'abonnement mensuel de Dermato Expert vous sera proposé à 39 € HT. Seriez-vous d'accord pour y souscrire ?*
[After the pilot, the monthly subscription for Dermato Expert will be offered at €39 excl. VAT. Would you agree to subscribe?]
**Response type:** Oui / Non (Yes / No) | **Included in:** Survey 2

**Q7.2:** *Nous travaillons un nouveau module d'IA qui permettrait d'évaluer la pathologie la plus probable et son niveau de sévérité, pour compléter Dermato Expert. Quel prix seriez-vous prêts à payer pour ce module supplémentaire ?*
[We are developing a new AI module to assess the most likely pathology and its severity level. What price would you be willing to pay for this additional module?]
**Response type:** Numeric (€/month) | **Included in:** Survey 2

## Notes

**Survey Administration:**

• Survey 1 (Preliminary): June 2024, 26 respondents out of 50 contracted pharmacies (52% response rate)

• Survey 2 (Feasibility): October-November 2024, 19 respondents out of 50 contracted pharmacies (38% response rate)

• Both surveys administered via Eval&Go professional online survey platform

**Response Rates:**

The lower response rate in Survey 2 compared to Survey 1 is attributable to survey fatigue, as pharmacies had already completed an initial survey only four months earlier. Importantly, satisfaction scores and reported trends remained consistent across both surveys, suggesting representative data.

**Question Variations:**

Some questions were specific to one survey or slightly modified between surveys. The 'Included in' column indicates which survey(s) contained each question.
